# Association between thyroid hormone sensitivity and carotid plaque risk: a health examination cohort-based study

**DOI:** 10.3389/fendo.2024.1472752

**Published:** 2024-12-11

**Authors:** Rui Gong, Shi Wang, Hongqiong Ding, Lixia Yu, Ming Xu, Sanping Xu, Yan Ling

**Affiliations:** ^1^ Health Management Center, Union Hospital, Tongji Medical College, Huazhong University of Science and Technology, Wuhan, China; ^2^ Department of Breast and Thyroid Surgery, Union Hospital, Tongji Medical College, Huazhong University of Science and Technology, Wuhan, China

**Keywords:** carotid plaque, thyroid hormone sensitivity, health examination, logistic regression, subgroup analysis

## Abstract

**Introduction:**

The involvement of thyroid hormone in cardiovascular disease remains debated. The aim of our research was to ascertain whether thyroid hormone sensitivity indices are related to carotid plaque (CAP) risk in the general population.

**Methods:**

We recruited 5,360 participants for health examinations to explore the correlation between thyroid hormone sensitivity indices and CAP risk. We then compared baseline characteristics of participants with CAP to those without CAP based on multivariate logistic regression analysis. Additionally, we conducted subgroup analyses stratified by gender and age to further elucidate this relationship.

**Results:**

Among the 5,360 participants, 1,055 (19.7%) were diagnosed with CAP. After adjusting for various confounding factors, our results showed a positive association between CAP risk and the indices (TFQI, PTFQI, TSHI, and TT4RI). Conversely, the FT3/FT4 ratio showed a negative correlation with CAP risk. Sex-based subgroup analysis revealed a stronger correlation between thyroid hormone sensitivity and CAP in females compared to males. In the age subgroup, the significant association was observed in older individuals (age >60) compared to middle-aged participants (age ≤60).

**Conclusion:**

Our study suggests a significant correlation between thyroid hormone sensitivity and CAP, particularly in females and participants over the age of 60.

## Introduction

Cardiovascular and cerebrovascular diseases have long posed significant challenges to global public health, with atherosclerosis-induced plaque formation being a critical pathophysiological mechanism (1, 2). Although atherosclerotic changes begin in childhood, symptoms typically do not appear until adulthood, around ages 40-45 in men and 55-60 in women (3, 4). Carotid plaque (CAP), as a hallmark of atherosclerosis, is defined by lipids accumulation, connective tissue within the carotid artery walls, and inflammatory cells infiltration (5). The presence of carotid plaques is closely linked to a higher risk of incidents related to heart and cerebral vessels, as well as overall mortality (6, 7). Studies have shown that carotid plaques (>2.6 mm) are present in 25% of 65-year-old men, with 2% having carotid stenosis (50-99%) (8). Thus, it is essential to comprehend the pathophysiology of CAP to identify and classify those who are at high risk for cardiovascular events early on (9, 10).

Thyroid hormones, particularly thyroxine (T4) and triiodothyronine (T3), are essential for metabolic regulation, cardiovascular function, and vascular health (11). In the context of carotid plaque, thyroid hormones influence lipid metabolism, endothelial function, and vascular remodeling, suggesting their involvement in atherosclerotic processes (12). Dysregulation of thyroid hormone levels, whether due to hypo- or hyperthyroidism, has been linked to various cardiovascular disorders, including hypertension, dyslipidemia, and atherosclerosis (13). The relationship between thyroid hormone abnormalities and atherosclerosis has garnered considerable attention.

Recent research indicated that thyroid hormones may significantly influence the occurrence and progression of atherosclerosis (14, 15). CAP, as an early indicator of atherosclerosis, is closely related to its pathogenesis. Previous studies have established a link between thyroid dysfunction and the onset and progression of CAP. For instance, Gu et al. found that longitudinal changes in thyroid function, particularly higher mean levels and greater fluctuations in thyroid hormones, were linked to an increased risk of developing carotid atherosclerosis (16). A study proposed that a serum TSH concentration ≥2.5 μIU/mL might be a potential indicator for evaluating the risk of atherosclerosis, especially in postmenopausal women (17). Another study observed that in individuals with coronary heart disease, greater sensitivity to both central and peripheral thyroid hormone is associated with an increased CAP risk, with more stronger associations in males, younger individuals, smokers, and drinkers (18). However, most previous research has primarily concentrated on measuring general thyroid hormone levels, such as T4 and TSH, and their direct effects on atherosclerosis. While these studies have provided valuable insights into the relationship between thyroid dysfunction and cardiovascular disease, they have not adequately addressed how the sensitivity to thyroid hormones influences the risk of developing CAP. Specifically, variations in thyroid hormone sensitivity may lead to differing impacts on cardiovascular health that are not captured by single hormone level alone.

This study systematically examines the association between physical examination indicators, particularly thyroid hormones sensitivity indices, and CAP risk across a diverse population. Additionally, our subgroup analyses based on age and sex will provide critical insights into how these demographic factors influence the relationship between thyroid sensitivity and CAP, ultimately offering tailored strategies for personalized interventions in clinical practice.

## Materials and methods

### Study population

Individuals who had a routine physical examination performed at the Health management Center of Wuhan Union Hospital from 2020 to 2023 were recruited for this study. The inclusion criteria were: (1) thyroid-related hormone testing; (2) carotid ultrasonography scanning measurements; (3) lipid metabolism parameters testing. The exclusion criteria included: (1) absence of TSH, FT3, FT4, or carotid ultrasound; (2) age < 18 years or > 75 years; (3) the existence of severe liver or renal illness, infectious diseases, cancer, or a history of thyroid surgery; (4) the use of medications that directly or indirectly affect thyroid hormone concentrations. Ultimately, 5360 participants were enrolled. Each participant provided written informed consent, and our hospital’s Ethics Committee approved the study procedure.

### Data collection

Physiological indicators (systolic blood pressure (SBP), diastolic blood pressure (DBP), weight, and height) were measured in accordance with established methods. Hematological and biochemical parameters, such as white blood cell count (WBC), platelet count (PLT), red blood cell count (RBC), alanine aminotransferase (ALT), aspartate aminotransferase (AST), triglycerides (TG), high-density lipoprotein cholesterol (HDL-C), creatinine (CREA), uric acid (UA), and fasting blood glucose (FBG) were detected by hematology and auto-biochemistry analyzers. Glycated hemoglobin (HbA1c), cystatin C (CysC), homocysteine (Hcy), thyroid peroxidase antibodies (TPOAb), thyroglobulin antibodies (TgAb), free thyroxine (FT4), thyroid-stimulating hormone (TSH), and free triiodothyronine (FT3) were analyzed by high-performance liquid chromatography and immunoassay analyzer. The criteria for dyslipidemia were defined as follows: triglycerides (TG) above 1.7 mmol/L, high-density lipoprotein cholesterol (HDL-C) below 1.0 mmol/L, low-density lipoprotein cholesterol (LDL-C) at or above 3.4 mmol/L, and total cholesterol (TC) at or above 5.2 mmol/L. Diabetes was diagnosed by fasting blood glucose (FBG) levels over 7.0 mmol/L, the use of diabetes medications, or a self-reported history of the disease. Hypertension was defined by the use of antihypertensive drugs, a systolic blood pressure (SBP) of 140 mmHg or more, or a diastolic blood pressure (DBP) of 90 mmHg or more.

Each participant underwent a carotid artery ultrasonographic examination, which was carried out by two highly skilled and experienced physicians using B-mode imaging and thorough scanning of the carotid arteries in multiple directions. The ultrasonographers were blinded to the clinical and laboratory data.

### Indices of thyroid hormone sensitivity

Thyroid hormone sensitivity was evaluated using both central and peripheral indices. Four different indices, namely TSH index (TSHI), TSH T4 resistance index (TT4RI), thyroid feedback quantile-based index (TFQI), and parametric thyroid feedback quantile-based index (PTFQI), were calculated to assess central sensitivity to thyroid hormones. Peripheral thyroid sensitivity was assessed using the FT3/FT4 ratio. For TFQI, PTFQI, TSHI, and TT4RI, higher values indicate lower central sensitivity to thyroid hormones, whereas higher FT3/FT4 values reflect greater peripheral sensitivity. The equations used for these calculations are as follows (17):


TSHII=lnTSH(miU/L)+0.1345 * FT4(pmol/L)

TT4RI=FTT4(pmol/L) * TSH(mIU/L)

TFQI=cdf fT4−(1−cdf TSH)

$$\begin{array}{c} \text{PTFQI}=\text{φ}((\text{fT}4-\text{μfT}4/\text{σfT}4)-(1-\text{φ}((\text{ln TSH}\\ -\text{μln TSH})/\text{σln TSH})) \end{array}$$

FT3/FT4 ratio=FT3(pmol/L)/FT4(pmol/L)

### Statistical analyses

Statistical analyses were performed using R software (version 4.3.1). Prior to analysis, data preprocessing involved handling missing values. Missing values for key variables (e.g., TSH, FT4), were imputed using multiple imputation methods. The Chi-square test was employed to compare categorical variables across groups, presented as numbers (%). Continuous variables were characterized using the median and interquartile range. Differences between groups were assessed by the independent samples t-test or the Mann-Whitney U test. To evaluate the association between CAP and thyroid hormone sensitivity indices, multivariate logistic regression was applied (17, 19). The odds ratios (ORs) and 95% confidence intervals (CIs) were calculated for each thyroid hormone index, adjusting for potential confounders. Three regression models were constructed: Model 1: Crude; Model 2: Adjusted for age and sex; Model 3: Adjusted for SBP, DBP, age, sex, HbA1c, HDL-C, LDL-C, TC, and TG. Subgroup analysis, adjusted for age/sex, SBP, DBP, TG, TC, HDL-C, and LDL-C, was used to examine assess the association between thyroid hormone sensitivity indices and the risk of CAP among gender (male/female), age (>60 years/≤60 years). A test for linear trend was conducted with the use of quartiles of the thyroid hormone sensitivity variable as a continuous variable by assigning the median values of the quartiles to the variable (20). Statistical significance was set at P < 0.05 (two-tailed).

## Results

### Characteristics of the study population

**Table 1** summarizes the baseline characteristics, showing that participants with CAP comprised 19.7%, with a higher incidence in male than in female. Compared to the non-CAP group, the CAP group’s individuals were significantly older and had higher levels of WBC, Hb, ALT, GGT, ALP, TG, LDL-C, BUN, CREA, UA, FBG, SBP, DBP, BMI, HbA1c, CysC, Hcy, ApoB, Lp(a), TFQI, and PTFQI. They also had significantly lower levels of HDL-C, ApoA1, and FT3. Additionally, the prevalence of type 2 diabetes, dyslipidemia, and hypertension is higher in the CAP group. No significant differences were observed in the level of RBC, TC, TPOAb, TgAb, FT4, and TSH between the two groups.

**Table 1:** Comparison of clinical characteristics.

Variable	Participants without CAP (N = 4305)	Participants with CAP (N =1055)	*P* value
Sex, Male/Female, %	2476/1829 (57.50/42.50)	839/216 (79.50/20.50)	<0.001
Age, median [IQR], y	50.00 [40.00, 55.00]	61.00 [54.00, 68.00]	<0.001
≤60/>60, y, %	3795/510 (88.20/11.80)	518/537 (49.10/50.90)	<0.001
WBC, median [IQR], 10^9/L	5.74 [4.89, 6.72]	5.99 [5.06, 7.20]	<0.001
Hb, median [IQR], g/dL	146.00 [134.00, 156.00]	148.00 [139.00, 157.00]	<0.001
PLT, median [IQR], 10^9/L	230.00 [194.00, 267.00]	218.00 [187.50, 254.00]	<0.001
RBC, median [IQR], 10^12/L	4.74 [4.40, 5.07]	4.75 [4.50, 5.02]	0.561
ALT, median [IQR], U/L	22.00 [15.00, 32.00]	23.00 [17.00, 33.00]	0.001
AST, median [IQR], U/L	24.00 [20.00, 30.00]	24.00 [21.00, 31.00]	<0.001
GGT, median [IQR], U/L	21.00 [14.00, 34.00]	24.00 [17.00, 39.00]	<0.001
ALP, median [IQR], U/L	66.00 [55.00, 79.00]	71.00 [60.00, 84.00]	<0.001
Dyslipidemia, No/Yes, %	2915/1390 (67.7/32.3)	601/454 (57.0/43.0)	<0.001
TG, median [IQR], mg/dL	1.25 [0.86, 1.94]	1.46 [1.01, 2.13]	<0.001
TC, median [IQR], mg/dL	4.93 [4.35, 5.58]	5.03 [4.30, 5.72]	0.053
HDL-C, median [IQR], mg/dL	1.33 [1.11, 1.63]	1.25 [1.03, 1.52]	<0.001
LDL-C, median [IQR], mg/dL	2.89 [2.40, 3.42]	3.02 [2.40, 3.63]	<0.001
BUN, median [IQR], mg/dL	4.67 [3.97, 5.48]	5.03 [4.30, 6.03]	<0.001
CREA, median [IQR], μmol/L	67.60 [57.20, 78.20]	73.40 [64.40, 83.10]	<0.001
UA, median [IQR], mg/dL	335.20 [268.70, 404.10]	357.10 [304.70, 422.10]	<0.001
Type 2 Diabetes, NO/Yes, %	4099/206 (95.2/4.8)	895/160 (84.8/15.2)	<0.001
FBG, median [IQR], mmol/L	4.90 [4.58, 5.30]	5.20 [4.80, 5.86]	<0.001
Hypertension, NO/Yes, %	2289/1531 (59.9/40.1)	321/621 (34.1/65.9)	<0.001
SBP, median [IQR], mmHg	118.00 [107.00, 130.00]	132.00 [120.00, 146.00]	<0.001
DBP, median [IQR], mmHg	74.00 [67.00, 82.00]	78.00 [70.00, 85.00]	<0.001
Height, median [IQR], cm	166.00 [160.00, 171.85]	167.00 [162.00, 172.00]	0.002
Weight, median [IQR], kg	65.60 [56.80, 75.00]	69.00 [62.00, 77.00]	<0.001
BMI, median [IQR], kg/m^2	23.80 [21.60, 26.30]	24.90 [22.80, 27.08]	<0.001
HbA1c, median [IQR], %	5.40 [5.20, 5.70]	5.70 [5.40, 6.00]	<0.001
CysC, median [IQR], mg/L	0.70 [0.63, 0.79]	0.80 [0.71, 0.92]	<0.001
Hcy, median [IQR], μmol/L	10.20 [8.20, 12.80]	12.00 [9.90, 15.20]	<0.001
TPOAb, median [IQR], IU/mL	1.00 [1.00, 1.47]	1.00 [1.00, 1.41]	0.45
ApoB, median [IQR], mg/dL	0.80 [0.67, 0.94]	0.86 [0.72, 1.05]	<0.001
ApoA1, median [IQR], mg/dL	1.40 [1.25, 1.59]	1.37 [1.23, 1.56]	0.014
Lp(a), median [IQR], mg/dL	11.30 [5.40, 25.10]	12.20 [6.40, 27.30]	0.037
TgAb, median [IQR], IU/mL	0.87 [0.62, 1.30]	0.84 [0.60, 1.25]	0.08
FT4, median [IQR], ng/dL	13.00 [12.20, 13.90]	13.10 [12.20, 14.00]	0.148
TSH, median [IQR], μIU/mL	1.75 [1.29, 2.37]	1.80 [1.29, 2.52]	0.122
FT3, median [IQR], pg/mL	4.60 [4.30, 4.90]	4.50 [4.20, 4.90]	0.01
TSHI, median [IQR]	2.32 [1.99, 2.64]	2.36 [2.00, 2.66]	0.052
TT4RI, median [IQR]	22.88 [16.67, 31.04]	23.29 [16.69, 32.38]	0.075
TFQI, median [IQR]	0.00 [-0.26, 0.28]	0.03 [-0.24, 0.31]	0.032
PTFQI, median [IQR]	-0.01 [-0.28, 0.26]	0.03 [-0.25, 0.30]	0.028
FT3/FT4, median [IQR]	0.35 [0.32, 0.38]	0.35 [0.32, 0.38]	0.006

### Relationship between thyroid hormone sensitivity and CAP

**Figure 1:**
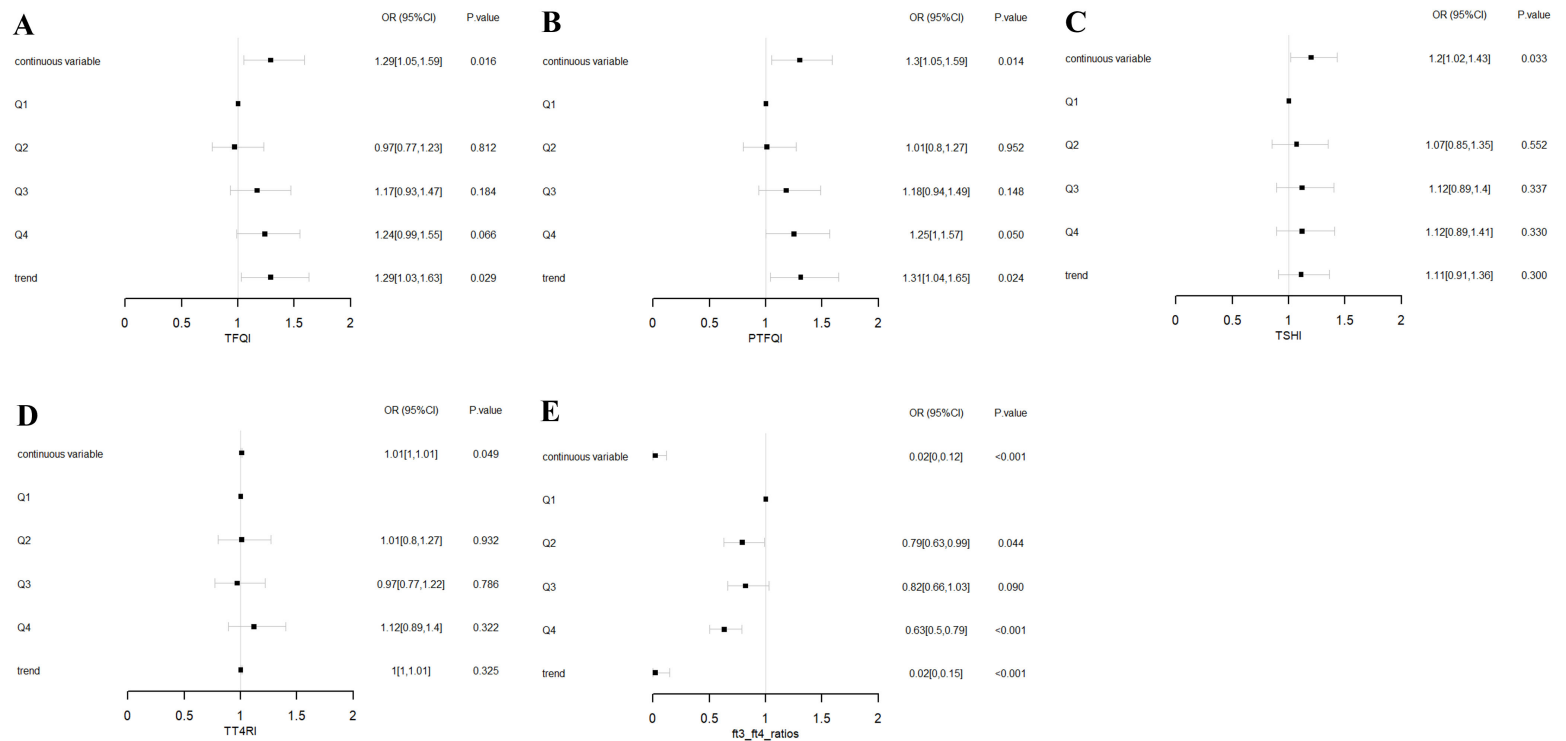
Forest plots of logistic regression analysis illustrating the association between thyroid hormone sensitivity and CAP. **(A)** ORs for CAP across TFQI quartiles. **(B)** ORs for CAP across PTFQI quartiles. **(C)** ORs for CAP across TSHI quartiles. **(D)** ORs for CAP across TT4RI quartiles. **(E)** ORs for CAP across FT3/FT4 ratios quartiles. Adjusted for age, sex, SBP, DBP, HbA1c, TC, TG, HDL-C, and LDL-C. Q1: first quartile; Q2: second quartile; Q3: third quartile; Q4: fourth quartile. P for trend based on variable containing median value for each quartile.

To assess the impact of thyroid hormone sensitivity on CAP, three logistic regression models were constructed (**Table 2** and **Figure 1**). In the multi-adjusted models, TFQI (OR: 1.29; 95% CI: 1.05–1.59; P = 0.016), PTFQI (OR: 1.30; 95% CI: 1.05–1.59; P = 0.014), TSHI (OR: 1.20; 95% CI: 1.02–1.43; P = 0.033), and TT4RI (OR: 1.01; 95% CI: 1.00–1.01; P = 0.049) were positively associated with CAP risk. In contrast, FT3/FT4 (OR: 0.02; 95% CI: 0.00–0.12; P < 0.001) showed a negative association with CAP, consistent with the results from the unadjusted model.

**Table 2:** Association between thyroid hormone sensitivity and CAP.

Variables	CAP					
	OR (95% CI)^1^	*P* value	OR(95% CI)^2^	*P* value	OR(95% CI)^3^	*P* value
TFQI	1.30 (1.08-1.58)	0.007	1.21 (1.02-1.44)	0.029	1.29 (1.05-1.59)	0.016
Q1	Reference		Reference		Reference	
Q2	0.97 (0.78-1.20)	0.750	0.97 (0.80-1.18)	0.765	0.97 (0.77-1.23)	0.812
Q3	1.20 (0.97-1.48)	0.097	1.15 (0.95-1.39)	0.159	1.17 (0.93-1.47)	0.184
Q4	1.26 (1.02-1.55)	0.034	1.18 (0.98-1.43)	0.082	1.24 (0.99-1.55)	0.066
P trend	1.33 (1.07-1.65)	0.011	1.23 (1.02-1.50)	0.034	1.29 (1.03-1.63)	0.029
PTFQI	1.30 (1.08-1.58)	0.006	1.22 (1.02-1.45)	0.025	1.30 (1.05-1.59)	0.014
Q1	Reference		Reference		Reference	
Q2	1.00 (0.81-1.25)	0.968	1.00 (0.82-1.22)	1.000	1.01 (0.80-1.27)	0.952
Q3	1.20 (0.98-1.49)	0.082	1.15 (0.95-1.39)	0.158	1.18 (0.94-1.49)	0.148
Q4	1.26 (1.02-1.56)	0.029	1.20 (0.99-1.45)	0.066	1.25 (1.00-1.57)	0.050
P trend	1.32 (1.07-1.64)	0.011	1.24 (1.02-1.51)	0.032	1.31 (1.04-1.65)	0.024
TSHI	1.24 (1.06-1.46)	0.007	1.19 (1.03-1.37)	0.020	1.20 (1.02-1.43)	0.033
Q1	Reference		Reference		Reference	
Q2	1.02 (0.82-1.26)	0.888	0.95 (0.79-1.16)	0.623	1.07 (0.85-1.35)	0.552
Q3	1.08 (0.88-1.34)	0.449	1.04 (0.86-1.26)	0.662	1.12 (0.89-1.40)	0.337
Q4	1.14 (0.92-1.41)	0.224	1.08 (0.89-1.30)	0.440	1.12 (0.89-1.41)	0.330
P trend	1.13 (0.94-1.36)	0.183	1.09 (0.92-1.29)	0.321	1.11 (0.91-1.36)	0.300
TT4RI	1.01 (1.00-1.02)	0.007	1.01 (1.00-1.01)	0.010	1.01 (1.00-1.01)	0.049
Q1	Reference		Reference		Reference	
Q2	0.98 (0.80-1.22)	0.881	0.95 (0.78-1.15)	0.589	1.01 (0.80-1.27)	0.932
Q3	0.98 (0.79-1.21)	0.859	0.96 (0.79-1.16)	0.659	0.97 (0.77-1.22)	0.786
Q4	1.15 (0.94-1.42)	0.180	1.13 (0.94-1.37)	0.197	1.12 (0.89-1.40)	0.322
P trend	1.01 (1.00-1.01)	0.147	1.01 (1.00-1.01)	0.129	1.00 (1.00-1.01)	0.325
FT3/FT4 ratio	0.03 (0.00-0.14)	<0.001	0.10 (0.02-0.45)	0.003	0.02 (0.00-0.12)	<0.001
Q1	Reference		Reference		Reference	
Q2	0.81 (0.65-1.00)	0.047	0.82 (0.68-0.99)	0.039	0.79 (0.63-0.99)	0.044
Q3	0.86 (0.70-1.06)	0.151	0.92 (0.76-1.10)	0.349	0.82 (0.66-1.03)	0.090
Q4	0.66 (0.53-0.81)	<0.001	0.75 (0.62-0.90)	0.003	0.63 (0.50-0.79)	<0.001
P trend	0.02 (0.00-0.19)	<0.001	0.09 (0.01-0.56)	0.010	0.02 (0.00-0.15)	<0.001

^1^crude model. ^2^adjusted for age and sex. ^3^adjusted for age, sex, SBP, DBP, HbA1c, TC, TG, HDL-C, and LDL-C.

### Subgroups analysis

The subgroup analyses by sex and age are presented in **Tables 3** and **4**. After adjusting for various potential confounders, we found that the FT3/FT4 ratio was negatively associated with CAP in both genders. Additionally, among females, significant correlations were observed between three thyroid hormones sensitivity indices (TFQI, PTFQI, and FT3/FT4 ratio) and CAP. Females had greater OR values than males. In the age subgroup analysis, participants aged up to 60 years showed a significant association with CAP only for the FT3/FT4 ratio. Conversely, among those aged over 60, TFQI and PTFQI demonstrated higher ORs for CAP risk, while the FT3/FT4 ratio displayed lower ORs.

**Table 3:** Subgroup analysis based on sex.

Sex	CAP						
	Variables	OR (95% CI)^1^	*P* value	OR (95% CI)^2^	*P* value	OR (95% CI)^3^	*P* value
**Female**	TFQI	1.7 (1.17-1.17)	0.005	1.58 (1.06-1.06)	0.0248	1.59 (1.04-1.04)	0.033
	Q1	Reference		Reference		Reference	
	Q2	1.13 (0.74-0.74)	0.567	1.04 (0.66-0.66)	0.878	1.00 (0.61-0.61)	0.984
	Q3	1.39 (0.92-0.92)	0.115	1.34 (0.86-0.86)	0.194	1.24 (0.78-0.78)	0.360
	Q4	1.53 (1.02-1.02)	0.041	1.38 (0.89-0.89)	0.152	1.34 (0.85-0.85)	0.210
	P trend	1.61 (1.06-1.06)	0.025	1.48 (0.94-0.94)	0.0921	1.43 (0.89-0.89)	0.142
	PTFQI	1.72 (1.19-1.19)	0.004	1.6 (1.07-1.07)	0.0222	1.62 (1.05-1.05)	0.028
	Q1	Reference		Reference		Reference	
	Q2	1.16 (0.75-0.75)	0.502	1.08 (0.68-0.68)	0.76	0.99 (0.60-0.60)	0.958
	Q3	1.52 (1.01-1.01)	0.048	1.44 (0.92-0.92)	0.11	1.32 (0.83-0.83)	0.241
	Q4	1.63 (1.09-1.09)	0.019	1.48 (0.95-0.95)	0.0837	1.43 (0.9-0.9)	0.128
	P trend	1.75 (1.15-1.15)	0.009	1.6 (1.01-1.01)	0.0452	1.56 (0.97-0.97)	0.071
	TSHI	1.57 (1.15-1.15)	0.004	1.40 (1.01-1.01)	0.0431	1.35 (0.95-0.95)	0.094
	Q1	Reference		Reference		Reference	
	Q2	1.15 (0.75-0.75)	0.510	1.29 (0.82-0.82)	0.274	1.30 (0.81-0.81)	0.282
	Q3	1.39 (0.92-0.92)	0.118	1.32 (0.85-0.85)	0.217	1.27 (0.79-0.79)	0.323
	Q4	1.50 (1.00-1.00)	0.052	1.33 (0.86-0.86)	0.201	1.28 (0.81-0.81)	0.298
	P trend	1.47 (1.03-1.03)	0.035	1.28 (0.87-0.87)	0.214	1.22 (0.81-0.81)	0.340
	TT4RI	1.02 (1.01-1.01)	0.004	1.01 (1.00-1.00)	0.0725	1.01 (1.00-1.00)	0.182
	Q1	Reference		Reference		Reference	
	Q2	1.29 (0.85-0.85)	0.237	1.43 (0.91-0.91)	0.121	1.49 (0.93-0.93)	0.097
	Q3	1.29 (0.85-0.85)	0.237	1.18 (0.75-0.75)	0.47	1.13 (0.70-0.70)	0.616
	Q4	1.59 (1.06-1.06)	0.025	1.45 (0.94-0.94)	0.0976	1.37 (0.87-0.87)	0.180
	P trend	1.02 (1.00-1.00)	0.032	1.01 (0.99-0.99)	0.195	1.01 (0.99-0.99)	0.381
	FT3/FT4 ratios	0.02 (0.00-0.00)	0.030	0.01(0-0)	0.00623	0.00 (0.00-0.00)	0.006
	Q1	Reference		Reference		Reference	
	Q2	0.82 (0.56-0.56)	0.309	0.91 (0.6-0.6)	0.648	0.92 (0.59-0.59)	0.712
	Q3	0.61 (0.4-0.4)	0.017	0.66 (0.43-0.43)	0.0687	0.67 (0.42-0.42)	0.084
	Q4	0.79 (0.53-0.53)	0.222	0.67 (0.44-0.44)	0.0657	0.64 (0.41-0.41)	0.049
	P trend	0.04 (0.00-0.00)	0.127	0.01 (0.00-0.00)	0.0354	0.01 (0.00-0.00)	0.026
**Male**	TFQI	1.12 (0.92-0.92)	0.273	1.21 (0.98-0.98)	0.08	1.22 (0.96-0.96)	0.102
	Q1	Reference		Reference		Reference	
	Q2	0.94 (0.75-0.75)	0.567	0.94 (0.74-0.74)	0.637	0.95 (0.73-0.73)	0.719
	Q3	1.05 (0.84-0.84)	0.682	1.09 (0.85-0.85)	0.505	1.06 (0.82-0.82)	0.649
	Q4	1.12 (0.90-0.90)	0.313	1.20 (0.95-0.95)	0.134	1.20 (0.93-0.93)	0.163
	P trend	1.15 (0.92-0.92)	0.218	1.24 (0.97-0.97)	0.081	1.24 (0.95-0.95)	0.119
	PTFQI	1.12(0.92-0.92)	0.263	1.21(0.98-0.98)	0.0788	1.22(0.96-0.96)	0.097
	Q1	Reference		Reference		Reference	
	Q2	0.99 (0.79-0.79)	0.909	0.99 (0.78-0.78)	0.961	1.00 (0.77-0.77)	0.990
	Q3	1.10 (0.88-0.88)	0.420	1.14 (0.9-0.9)	0.284	1.15 (0.88-0.88)	0.299
	Q4	1.13 (0.90-0.90)	0.285	1.21 (0.95-0.95)	0.118	1.21 (0.93-0.93)	0.149
	P trend	1.16 (0.92-0.92)	0.203	1.25 (0.98-0.98)	0.0714	1.25 (0.96-0.96)	0.098
	TSHI	1.21 (1.02-1.02)	0.026	1.19 (1.00-1.00)	0.057	1.18 (0.97-0.97)	0.106
	Q1	Reference		Reference		Reference	
	Q2	0.90 (0.72-0.72)	0.363	0.92 (0.73-0.73)	0.527	0.96 (0.73-0.73)	0.752
	Q3	0.88 (0.70-0.70)	0.259	0.84 (0.66-0.66)	0.153	0.84 (0.64-0.64)	0.199
	Q4	1.15 (0.93-0.93)	0.204	1.13 (0.89-0.89)	0.315	1.13 (0.87-0.87)	0.349
	P trend	1.12 (0.92-0.92)	0.242	1.09 (0.88-0.88)	0.451	1.08 (0.85-0.85)	0.520
	TT4RI	1.01 (1.00-1.00)	0.005	1.01 (1.00-1.00)	0.0439	1.01 (1.00-1.00)	0.116
	Q1	Reference		Reference		Reference	
	Q2	0.89 (0.71-0.71)	0.304	0.89 (0.70-0.70)	0.362	0.91 (0.70-0.70)	0.505
	Q3	0.88 (0.70-0.70)	0.258	0.85 (0.66-0.66)	0.176	0.83 (0.64-0.64)	0.173
	Q4	1.19 (0.96-0.96)	0.110	1.12 (0.88-0.88)	0.35	1.11 (0.85-0.85)	0.442
	P trend	1.01 (1.00-1.00)	0.055	1.01 (1.00-1.00)	0.258	1.00 (0.99-0.99)	0.382
	FT3/FT4 ratios	0.01 (0.00-0.00)	<0.001	0.03 (0.00-0.00)	0.000423	0.03 (0.00-0.00)	0.001
	Q1	Reference		Reference		Reference	
	Q2	0.83 (0.67-0.67)	0.096	0.88 (0.70-0.70)	0.298	0.87 (0.67-0.67)	0.277
	Q3	0.75 (0.60-0.60)	0.009	0.86 (0.68-0.68)	0.222	0.85 (0.66-0.66)	0.227
	Q4	0.60 (0.48-0.48)	<0.001	0.70 (0.55-0.55)	0.00382	0.70 (0.54-0.54)	0.009
	P trend	0.01 (0.00-0.00)	<0.001	0.04 (0.00-0.00)	0.00464	0.04 (0.00-0.00)	0.011

^1^crude model. ^2^adjusted for age. ^3^adjusted for age, SBP, DBP, TG, TC, HDL-C, and LDL-C.

**Table 4:** Subgroup analysis based on age.

Age	CAP						
	Variables	OR (95% CI)^1^	*P* value	OR (95% CI)^2^	*P* value	OR (95% CI)^3^	*P* value
**≤60**	TFQI	1.22 (0.96-0.96)	0.102	1.21 (0.95-0.95)	0.119	1.21 (0.93-0.93)	0.157
	Q1	Reference		Reference		Reference	
	Q2	0.99 (0.76-0.76)	0.952	0.98 (0.75-0.75)	0.897	1.04 (0.77-0.77)	0.789
	Q3	1.12 (0.87-0.87)	0.383	1.13 (0.86-0.86)	0.379	1.10 (0.82-0.82)	0.540
	Q4	1.17 (0.90-0.90)	0.233	1.16 (0.89-0.89)	0.267	1.14 (0.86-0.86)	0.358
	P trend	1.21 (0.93-0.93)	0.163	1.2(0.92-0.92)	0.183	1.16 (0.86-0.86)	0.329
	PTFQI	1.23 (0.97-0.97)	0.088	1.22 (0.96-0.96)	0.103	1.22 (0.94-0.94)	0.139
	Q1	Reference		Reference		Reference	
	Q2	1.05 (0.80-0.80)	0.728	1.06 (0.81-0.81)	0.675	1.11 (0.82-0.82)	0.492
	Q3	1.17 (0.90-0.90)	0.228	1.20 (0.92-0.92)	0.187	1.18 (0.88-0.88)	0.257
	Q4	1.18 (0.91-0.91)	0.204	1.18 (0.91-0.91)	0.215	1.17 (0.88-0.88)	0.288
	P trend	1.22 (0.93-0.93)	0.15	1.21 (0.93-0.93)	0.156	1.18 (0.88-0.88)	0.262
	TSHI	1.17 (0.96-0.96)	0.128	1.26 (1.03-1.03)	0.023	1.21 (0.97-0.97)	0.093
	Q1	Reference		Reference		Reference	
	Q2	1.11 (0.86-0.86)	0.426	1.14 (0.88-0.88)	0.329	1.25 (0.94-0.94)	0.126
	Q3	0.93 (0.71-0.71)	0.592	0.99 (0.76-0.76)	0.955	0.97 (0.72-0.72)	0.851
	Q4	1.09 (0.84-0.84)	0.505	1.19 (0.92-0.92)	0.193	1.18 (0.89-0.89)	0.260
	P trend	1.03 (0.82-0.82)	0.773	1.12 (0.89-0.89)	0.326	1.09 (0.84-0.84)	0.529
	TT4RI	1.00 (1.00-1.00)	0.316	1.01 (1.00-1.00)	0.039	1.01 (1.00-1.00)	0.168
	Q1	Reference		Reference		Reference	
	Q2	1.03 (0.79-0.79)	0.837	1.05 (0.81-0.81)	0.711	1.08 (0.81-0.81)	0.601
	Q3	0.93 (0.71-0.71)	0.596	0.99 (0.76-0.76)	0.946	0.93 (0.69-0.69)	0.632
	Q4	1.06 (0.82-0.82)	0.64	1.20 (0.92-0.92)	0.177	1.14 (0.86-0.86)	0.363
	P trend	1.00 (0.99-0.99)	0.734	1.01 (1.00-1.00)	0.201	1.00 (0.99-0.99)	0.480
	FT3/FT4 ratios	1.33 (0.16-0.16)	0.788	0.10 (0.01-0.01)	0.039	0.05 (0.00-0.00)	0.012
	Q1	Reference		Reference		Reference	
	Q2	0.81 (0.62-0.62)	0.128	0.74 (0.56-0.56)	0.032	0.70 (0.52-0.52)	0.020
	Q3	1.14 (0.88-0.88)	0.318	0.95 (0.73-0.73)	0.685	0.90 (0.68-0.68)	0.466
	Q4	1.03 (0.79-0.79)	0.845	0.76 (0.58-0.58)	0.042	0.67 (0.50-0.50)	0.008
	P trend	2.99 (0.24-0.24)	0.393	0.14 (0.01-0.01)	0.139	0.05 (0.00-0.00)	0.033
**>60**	TFQI	1.34 (0.98-0.98)	0.070	1.45 (1.05-1.05)	0.023	1.50 (1.06-1.06)	0.021
	Q1	Reference		Reference		Reference	
	Q2	0.91 (0.65-0.65)	0.600	0.94 (0.66-0.66)	0.722	0.96 (0.67-0.67)	0.840
	Q3	1.23 (0.87-0.87)	0.237	1.30 (0.91-0.91)	0.145	1.36 (0.94-0.94)	0.105
	Q4	1.32 (0.93-0.93)	0.116	1.43 (1.01-1.01)	0.046	1.49 (1.02-1.02)	0.038
	P trend	1.42 (1.00-1.00)	0.051	1.55 (1.08-1.08)	0.017	1.63 (1.10-1.10)	0.014
	PTFQI	1.32 (0.97-0.97)	0.078	1.44 (1.04-1.04)	0.026	1.50 (1.06-1.06)	0.022
	Q1	Reference		Reference		Reference	
	Q2	0.86 (0.61-0.61)	0.382	0.88 (0.62-0.62)	0.477	0.90 (0.62-0.62)	0.560
	Q3	1.17 (0.83-0.83)	0.358	1.26 (0.89-0.89)	0.200	1.30 (0.90-0.90)	0.168
	Q4	1.30 (0.92-0.92)	0.137	1.40 (0.99-0.99)	0.059	1.47 (1.01-1.01)	0.043
	P trend	1.41 (0.99-0.99)	0.060	1.53 (1.07-1.07)	0.021	1.62 (1.10-1.10)	0.015
	TSHI	1.10 (0.85-0.85)	0.474	1.20 (0.93-0.93)	0.167	1.24 (0.94-0.94)	0.127
	Q1	Reference		Reference		Reference	
	Q2	0.82 (0.58-0.58)	0.256	0.82 (0.58-0.58)	0.256	0.88 (0.61-0.61)	0.488
	Q3	1.07 (0.76-0.76)	0.691	1.14 (0.8-0.8)	0.463	1.28 (0.88-0.88)	0.196
	Q4	0.97 (0.69-0.69)	0.861	1.07 (0.75-0.75)	0.705	1.14 (0.79-0.79)	0.483
	P trend	1.03 (0.77-0.77)	0.819	1.14 (0.84-0.84)	0.404	1.22 (0.89-0.89)	0.226
	TT4RI	1.00 (0.99-0.99)	0.359	1.01 (1.00-1.00)	0.096	1.01 (1.00-1.00)	0.116
	Q1	Reference		Reference		Reference	
	Q2	0.96 (0.68-0.68)	0.793	0.96 (0.68-0.68)	0.804	1.05 (0.72-0.72)	0.806
	Q3	0.88 (0.62-0.62)	0.458	0.94 (0.66-0.66)	0.733	1.03 (0.71-0.71)	0.881
	Q4	1.02 (0.72-0.72)	0.93	1.13 (0.80-0.80)	0.490	1.18 (0.81-0.81)	0.394
	P trend	1.00 (0.99-0.99)	0.948	1.00 (0.99-0.99)	0.448	1.01 (0.99-0.99)	0.405
	FT3/FT4 ratios	0.01 (0.00-0.00)	<0.001	0.00 (0.00-0.00)	<0.001	0.00 (0.00-0.00)	<0.001
	Q1	Reference		Reference		Reference	
	Q2	0.84 (0.60-0.60)	0.328	0.79 (0.56-0.56)	0.192	0.81 (0.56-0.56)	0.282
	Q3	0.72 (0.51-0.51)	0.0605	0.65 (0.45-0.45)	0.016	0.68 (0.46-0.46)	0.042
	Q4	0.52 (0.37-0.37)	<0.001	0.48 (0.34-0.34)	<0.001	0.54 (0.37-0.37)	0.002
	P trend	0.00 (0.00-0.00)	<0.001	0.00 (0.00-0.00)	<0.001	0.00 (0.00-0.00)	0.001

^1^crude model. ^2^adjusted for sex. ^3^adjusted for sex, SBP, DBP, TG, TC, HDL-C, and LDL-C.

## Discussion

This cross-sectional study observed a significant increase in various physiological indicators such as age, WBC, Hb, ALT, GGT, ALP, and TG among individuals with CAP compared to those without CAP. multivariate logistic regression analyses indicated that central thyroid hormone sensitivity indices were linked to an elevated risk of CAP, whereas the FT3/FT4 ratio was associated with a decreased risk Subgroup analysis further highlighted these differences, particularly among females and individuals over 60 years old.

Previous studies investigating the association between thyroid hormone levels or thyroid dysfunction and CAP have produced conflicting results, which may be attributed to differences in study populations and methodologies. For instance, Delitala et al. conducted research in Italy, identifying carotid plaques using subjective criteria, defined as focal encroachments of the arterial wall. They found no correlation between thyroid hormone levels and increased intima-media thickness (IMT) or the presence of CAP, concluding that thyroid hormone levels did not predict carotid atherosclerosis after adjusting for confounding variables (18). Similarly, Kim et al. studied a Korean population, using carotid duplex ultrasonography (DUS) with a 4.4-MHz pulsed Doppler device to detect CAP, which was defined as focal carotid intima-media thickness (IMT) greater than 1.5 mm or vessel wall thickening by at least 50% relative to the surrounding wall. Their findings supported the notion that persistent subclinical thyroid dysfunction did not influence the baseline presence or progression of CAP in healthy individuals (21). On the other hand, Völzke et al. examined German participants aged 45 years and older, using ultrasound to assess carotid IMT, and suggested that increased IMT might be independently associated with thyroid function (22). Additionally, research from Pomerania focused on individuals aged 45 and older, assessing the presence of carotid plaques with B-mode ultrasound and reporting a higher prevalence of CAP linked to lower TSH levels (14). These variations in population characteristics, such as age and region, along with differences in the methods for detecting and defining CAP, could account for the inconsistencies observed across studies. For example, the subjectivity in plaque identification or differences in ultrasound technology and analysis techniques might influence the observed associations. Furthermore, the potential influence of unmeasured confounders and varying durations of thyroid dysfunction could also contribute to these conflicting results, underscoring the complexity of the relationship between thyroid hormone levels and CAP and the need for further research.

In order to minimize deviations and averting severe results in thyroid dysfunction instances, Laclaustra et al. introduced new indices that will provide a more accurate representation of the relationships between changes in thyroid hormones and CAP (23). Furthermore, Liu et al. found that TSHI, TT4RI, PTFQI, and FT3/FT4 indices were associated with the risk of CAP in the coronary heart disease (CHD) population (17). In our study, regardless of sex and age, our findings in the general population were consistent with those in the CHD population.

Notably, sex and age-related differences in the relationship between thyroid hormone sensitivity and CAP risk may offer crucial insights into the underlying endocrine mechanisms. The stronger correlation between thyroid hormone sensitivity indices (TFQI, PTFQI, and FT3/FT4 ratio) and CAP in females suggests a potentially heightened vulnerability or a distinct pathophysiological response to thyroid hormone fluctuations compared to males (24). This heightened sensitivity in females, reflected by higher OR values, may be attributed to hormonal differences, possibly involving estrogen, which is known to influence thyroid function and immune responses (25, 26). Additionally, the age subgroup analysis revealed a dynamic shift in thyroid hormone sensitivity’s impact on CAP risk (15). Age below 60, only FT3/FT4 ratio was a significant predictor, while age over 60, TFQI and PTFQI emerged as stronger predictors, suggesting that with advancing age, broader thyroid dysfunction becomes more relevant in influencing CAP risk. This differential impact underscores the importance of tailored approaches in assessing thyroid function and managing CAP risk across different demographic groups.

Potential mechanisms linking thyroid hormone sensitivity to carotid plaque (CAP) development may involve the hormone’s effects on lipid metabolism, endothelial function, and inflammatory pathways, all of which contribute to atherosclerosis. For example, increased levels of free triiodothyronine (FT3) are linked to a lower likelihood of elevated total cholesterol (TC) and LDL-C, and there is a minor positive association between TSH and decreased HDL-C (27). Higher TSH or lower FT4 are causally linked to elevated total cholesterol and LDL, with no evidence of a reverse causal relationship (28). This lipid imbalance promotes cholesterol accumulation in arterial walls, contributing to the formation of atherosclerotic plaques (29). In our study, we also found that patients with CAP exhibited lower HDL-C and higher LDL-C levels compared to healthy individuals. Additionally, thyroid hormones influence inflammatory processes and endothelial function. Altered thyroid hormone sensitivity may increase the expression of pro-inflammatory cytokines and impair vasodilation, further promoting atherosclerosis. Consequently, dysregulated thyroid hormone activity can drive both lipid accumulation and inflammation in the arteries, heightening the risk of carotid plaque formation and subsequent cardiovascular events. Theoretically, this suggests that individuals with altered thyroid hormone sensitivity may be at higher risk for developing carotid plaques due to the dysregulation of these pathways. Practically, our findings emphasize the potential value of incorporating thyroid function assessments into cardiovascular risk stratification. Evaluating thyroid hormone levels in patients with atherosclerotic disease could guide personalized treatment strategies to reduce plaque burden and improve vascular health. Future research should delve deeper into the mechanisms by which thyroid hormones influence carotid plaque formation and explore the therapeutic potential of thyroid hormone modulation in preventing or treating atherosclerosis.

However, this study has several limitations. Firstly, despite its large scale, our study’s cross-sectional design inherently limits the ability to establish a causal relationship between thyroid hormone sensitivity and the progression of CAP. Additionally, we cannot exclude the possibility of reverse causality, where thyroid function could be influenced by CAP. Secondly, although we adjusted for several potential confounders, we acknowledge that lifestyle factors, such as smoking habits, physical activity, and dietary patterns, were not considered. These unmeasured factors have influenced the observed associations between cardiovascular health and thyroid function. Thirdly, recruiting participants from a single-center health examination cohort may have introduced selection bias, limiting the external validity of our findings. Fourthly, the study’s focus on Chinese individuals may introduce racial disparities, potentially limiting the generalizability of the findings to other populations. Consequently, well-conducted randomized controlled trials are necessary to further validate our findings and establish causal relationships.

In this study, we found that thyroid hormone sensitivity indices are significantly linked to the CAP risk in the general population. Subgroup analysis revealed a stronger association in older participants (age >60) and females compared to younger participants (age ≤ 60) and males. This study provides reliable evidence that can enhance prevention strategies and clinical treatment for individuals with CAP.

## Data Availability

The raw data supporting the conclusions of this article will be made available by the authors, without undue reservation.
